# A Novel Approach to Derive the Predicted No-Effect Concentration (PNEC) of Benzophenone-3 (BP-3) Using the Species Sensitivity Distribution (SSD) Method: Suggestion of a New PNEC Value for BP-3

**DOI:** 10.3390/ijerph18073650

**Published:** 2021-03-31

**Authors:** Jae-Woong Jung, Jae Soon Kang, Jinsoo Choi, June-Woo Park

**Affiliations:** 1 Center for Defense Acquisition and Requirements Analysis, Korea Institute for Defense Analyses, Seoul 02455, Korea; jwjung@kida.re.kr; 2 Department of Anatomy and Convergence Medical Science, Institute of Health Science, Bio Anti-Aging Medical Research Center, Gyeongsang National University Medical School, Jinju 52727, Korea; jskang@gnu.ac.kr; 3 Department of Environmental Toxicology and Chemistry, Korea Institute of Toxicology, Jinju 52834, Korea; jschoi1020@kitox.re.kr; 4 Human and Environmental Toxicology Program, Korea University of Science and Technology (UST), Daejeon 34113, Korea

**Keywords:** benzophenone-3, species sensitivity distribution, chronic toxicity, risk assessment, freshwater

## Abstract

The necessity for the aquatic ecological risk assessment for benzophenone-3 (BP-3) is increasing due to its high toxic potential and high detection frequency in freshwater. The initial step in the ecological risk assessment is to determine predicted no-effect concentration (PNEC). This study derived PNEC of BP-3 in freshwater using a species sensitivity distribution (SSD) approach, whilst existing PNECs are derived using assessment factor (AF) approaches. A total of eight chronic toxicity values, obtained by toxicity testing and a literature survey, covering four taxonomic classes (fish, crustaceans, algae, and cyanobacteria) were used for PNEC derivation. Therefore, the quantity and quality of the toxicity data met the minimum requirements for PNEC derivation using an SSD approach. The PNEC derived in this study (73.3 μg/L) was far higher than the environmental concentration detected in freshwater (up to 10.4 μg/L) as well as existing PNECs (0.67~1.8 μg/L), mainly due to the difference in the PNEC derivation methodology (i.e., AF vs. SSD approach). Since the SSD approach is regarded as more reliable than the AF approach, we recommend applying the PNEC value derived in this study for the aquatic ecological risk assessment of BP-3, as the use of the existing PNEC values seems to unnecessarily overestimate the potential ecological risk of BP-3 in freshwater.

## 1. Introduction

Benzophenones (BPs) have ultraviolet protective properties; therefore, they have been frequently utilized as photo initiators, ultraviolet (UV) blockers, and photophysical probes in several industrial applications and personal care products [1]. Benzophenone-3 (BP-3) is one of the most widely used BPs, common in plastics, sunscreens, and cosmetics. Its ubiquity means it can be readily introduced to freshwater systems directly from water recreational activities (e.g., removal from human skin during swimming and bathing) and indirectly from sewage discharge (e.g., the release of wastewater originating from showering and/or laundry processes) [2,3]. Benzophenones, including BP-3, are considered pseudo-persistent pollutants due to their extensive use and continuous disposal [4].

BP-3 is detected in the environment in the range of ng/L to μg/L [1]. Since high concentrations of BP-3 have been reported around wastewater treatment plants (WWTPs), WWTP effluent has been considered the main source of pollutant BP-3 [5]. In freshwater, BP-3 is usually detected at concentrations in the low ng/L range, but in some extreme cases, it has been detected at very high concentrations of hundreds to thousands of ng/L [6,7]. BP-3 has high stability due to its low susceptibility to photolysis, and it has been detected in various environments [1]. In addition, it is known that BP-3 can bio-accumulate [8], so even though its concentration in the environment may be low, the toxicity of BP-3 is expected to be high.

In vitro experiments have shown BP-3 to have slight or moderate estrogenic toxicity [9,10]. BP-3 is easily metabolized to other analogues, such as BP-1 and BP-8 [4]; of note, it has been reported that BP-1 demonstrated higher estrogenic toxicity than BP-3 in yeast two-hybrid assays [11]. In addition, BP-3 has been proven to cause acute toxicity and estrogenic toxicity in many aqueous organisms. In acute toxicity tests on aqueous organisms such as algae, water flea, and fish, BP-3 showed a median effective concentration (EC50) or a median lethal concentration (LC50) at the mg/L concentration level [12,13]. BP-3 induces estrogenic and reproductive toxicity by endocrine disruption in fish [6,14,15,16].

These previous toxicity and environmental monitoring studies clearly demonstrated that BP-3 can provoke potential risk to aquatic species. The initial step in the ecological risk characterization of a chemical is to determine the predicted no-effect concentration (PNEC) and compare it to the detected concentration [17]. If the detected concentration of a chemical is lower than the PNEC value, it is considered that the chemical will not cause any adverse effects on the ecosystem. In contrast, if the detected concentration of a chemical exceeds the PNEC value, it is concluded that the chemical may have adverse effects on the ecosystem, and thus, risk management action and/or further detailed assessment is warranted. Two approaches have been proposed to derive the PNEC value of a chemical: the assessment factor (AF) approach and the species sensitivity distribution (SSD) approach. The AF approach derives a PNEC by dividing the lowest toxicity value (e.g., median lethal concentration and no observed effect concentration) of a chemical by an assessment factor (e.g., 10, 100, or 1000) assigned according to the toxicity data available. On the other hand, in the SSD approach, the statistical distribution of toxicity data of a chemical is determined, and then the PNEC value is set at the desired safe level (e.g., 5th percentile) of the distribution.

To the best of our knowledge, three PNECs for BP-3 have been suggested using AF approaches, ranging from 0.67~1.8 μg/L [4,12,18]. For example, Kim and Choi [4] derived the PNEC of BP-3 (i.e., 1.32 μg/L) by dividing the lowest chronic toxicity value (132 μg/L) by an assessment factor of 100. However, the SSD approach has been suggested as a more reliable tool than the AF approach because the SSD approach employs more toxicity data, while the AF approach derives the PNEC value from only limited toxicity data [19]. For this reason, in several countries, including Australia and New Zealand, the SSD approach is preferred to the AF approach when deriving the PNEC value of a chemical [20]. Hence, it is better to derive the PNEC value of BP-3 using the SSD approach, if possible.

To derive a reliable PNEC value for a specific chemical using the SSD approach, the minimum toxicity data requirements (at least eight chronic toxicity datasets from at least four taxonomic classes) must be met [20]. Earlier studies on BP-3 were compelled to derive the PNEC using an AF approach, presumably due to the lack of chronic toxicity data. For example, Du, Wang, Pei, Ahmad, Xu, Zhang and Sun [12] calculated the PNEC of BP-3 using both the AF and SSD approaches, but they disregarded the value obtained from the SSD approach as the toxicity data were insufficient (toxicity data from six species covering only three taxonomic classes), so the PNEC value derived using the SSD approach was unreliable. Therefore, it is a prerequisite to collect toxicity data covering four or more taxonomic classes to derive the PNEC of BP-3 using the SSD approach.

The aim of the present study was to suggest a value of freshwater PNEC of BP-3 using the SSD approach. For this, chronic aquatic toxicity data were obtained through toxicity data collection and toxicity testing. In this study, chronic toxicity tests for three aquatic species were conducted, including a fish (*Cyprinus carpio*), crustacean (*Moina macrocopa*), and green alga (*Pseudokirchneriella subcapitata*) species. To the best of our knowledge, this study is the first to calculate the freshwater PNEC of BP-3 using the SSD approach, which seems to be more statistically powerful than AF approach.

## 2. Materials and Methods

### 2.1. BP-3 Toxicity to Aqueous Organisms

#### 2.1.1. BP-3

BP-3 (CAS NO. 131-57-7, 98%) used in this study was purchased from Sigma-Aldrich (St. Louis, MI, USA). Dimethylformamide (DMF, Sigma-Aldrich, St. Louis, MI, USA) was used as a solvent to enhance solubility of BP-3 in the tested media.

#### 2.1.2. Aqueous Organisms

In this study, three Korean resident species were used to produce acute and chronic toxicological data for the development of water quality criteria in Korea (WQCKOREA): *P. subcapitata* (an alga), *M. macrocopa* (a crustacean), and *C. carpio* (a fish). *P. subcapitata* was obtained from the algae culture facility of the Korea Institute of Toxicology (KIT, Jinju, Korea) and cultivated in American Type Culture Collection (ATCC) medium at 22 ± 2 °C. The biological activity of the algae used in the test was maintained as follows: algal cells were transplanted into a solid medium (i.e., agar-amended ATCC medium) at least once every other month to form single cells. The selected single cells were then cultured in ATCC medium; cells were sub-cultured once per week. *M. macrocopa* were supplied by the National Institute of Environmental Research (Incheon, Korea) and cultivated in M4 media at 20 ± 2 °C according to the method recommended by the Organization for Economic Cooperation and Development (OECD). Juvenile *C. carpio*, with a body length of 2.5 ± 0.5 cm, were purchased from a commercial fish farm (Ochang fish farm, Ochang, Korea) and acclimated for 2 weeks at 25 ± 2 °C and in a 12:12 h light/dark cycle before use in the experiment.

#### 2.1.3. Determination of Solubility in the Test Media

Since BP-3 is not soluble in water, its solubility was measured in each medium (ATCC, M4 and fish water). Briefly, 100 mg BP-3 was added to each 2 L medium and the mixture was stirred using a magnetic bar for 1 h at 24 ± 2 °C. After stirring, the solution was allowed to stand for 24 h at 24 ± 2 °C. All solutions above 1/3 of the beaker bottom were carefully collected, and the concentration of BP-3 in each medium was measured using high-performance liquid chromatography (HPLC) at the Center for Research Faculties of Gyeongsang National University (CRF-GNU, Jinju, Korea). The concentration measured in the solution was determined as the maximum concentration (C0) in each medium. The C0 values for each medium are summarized in Table 1.

#### 2.1.4. Acute Toxicity

Acute toxicity tests of *P. subcapitata*, *M. macrocopa*, and *C. carpio* were performed according to protocols recommended by the OECD (OECD test guidelines 201, 202 and 203, respectively) [21,22,23]. The aqueous species were exposed to one-fifth concentration of C0 (Table 1). No solvent chemical was used for the acute toxicity tests. The endpoints for each of the investigations were: growth inhibition in the algae toxicity test, immobilization in the water flea toxicity test, and mortality in the fish toxicity test. Medium without BP-3 was used as a control. Each toxicity endpoint (EC50 or LC50) was calculated using the Comprehensive Environmental Toxicity Information System™ (CETIS, Tidepool Scientific Software, McKinleyville, CA, USA). Acute toxicity tests on *P. subcapitata* and *M. macrocopa* were performed in three and four replicates, respectively. An acute toxicity test was performed on *C. carpio* without replication.

#### 2.1.5. Chronic Toxicity

The chronic toxicity tests were performed for *M. macrocopa* and *C. carpio* using the methods described in a previous study [24] and the OECD test guideline 229 [25], respectively. Based on the toxicity data from the acute toxicity tests, *M. macrocopa* and *C. carpio* were exposed to several concentrations of BP-3, respectively (Table 1). Medium (M4 and fish water) without chemicals was used as a blank control, and medium with 0.01% DMF was used as a solvent control, which helps to maintain the designated concentrations of BP3 in the media during a given exposure time. 

In the chronic toxicity test of *M. macrocopa*, the healthy neonates (<24 h old) were randomly selected and individually placed in 30 mL medium with or without chemical in 50 mL glass beakers (10 neonates/group), and then incubated at 20 ± 2 °C for 14 days. During the test, fresh chlorella was fed to *M. macrocopa* once daily and test subjects were transferred to fresh medium every other day to minimize any decrease in chemical concentration. Survival and reproduction (first day of reproduction, number of young per female, and brood size) were observed daily as toxicity endpoints. 

In the chronic toxicity test of *C. carpio*, healthy, similarly sized *C. carpio* individuals were randomly selected, and five fish were placed in 1.5 L medium in 2 L glass beakers. Fish were exposed to 5 concentrations of BP-3 at 25 ± 2 °C for 21 days. During the test, fresh artemia (<24 h old) was fed to fish twice daily. To prevent ammonia toxicity, dissolved oxygen depletion, and chemical concentration reduction, half of the medium was exchanged 3 times a week, and the medium was replaced completely once a week. This test was performed in triplicate. During the test, the survival and behavior were observed daily, and no abnormal behavior was observed. On the last day, the fish were anesthetized by cold shock using ice. The livers were then collected by dissection, immediately soaked in RNAlater (Sigma-Aldrich) and stored at 4 °C until use. The livers were crushed using TissueLyser II (Qiagen, Hilden, Germany), from which total RNA was extracted using an RNeasy Plus Mini Kit (Qiagen). RNA was quantified using a Synergy H1 microplate reader (Biotek, Winooski, VT, USA) and all RNA quality (260/280 ratio) was confirmed to be between 1.9 and 2.1. Complementary DNA (cDNA) was synthesized from the total RNA using Superscript^®^ VI First-Strand Synthesis System (Thermo Fisher Scientific, Waltham, MA, USA). Primer information for vitellogenin 1 and 18S ribosomal RNA genes (vtg1 and 18S rRNA) for quantitative real-time PCR (qPCR) is summarized in Table S1 of the Supplementary Materials. The efficiency of all primers was between 90% and 100% (−3.6 ≥ slope ≥ −3.3). The qPCR mixture was composed of 10 μL 2 × GO Taq Master Mix (Promega Corporation, Fitchburg, WI, USA), 5 pmol primers, and 100 ng cDNA. qPCR was conducted under the following conditions: 40 cycles of 95 °C/20 s, 58 °C/20 s, and 72 °C/20 s using an Mx3000 qPCR system (Agilent, Santa Clara, CA, USA). The relative transcriptional levels were calculated using the 2-ΔCt method. 

Since 72 h (or longer) no observed effect concentration (NOEC) values can be used as a chronic result [17], the 72 h-NOEC value of *P. subcapitata* was directly derived from the acute toxicity test result, instead of conducting another chronic test.

#### 2.1.6. Quantification of BP-3 and Ammonia in the Tested Medium

During the chronic toxicity test of *C. carpio*, when the culture medium was changed (three times a week), the medium (50 mL) was collected before and after replacement in the highest concentration medium (0.6 mg/L), and BP-3 was quantified using HPLC in CFR-GNU. The ammonia concentration was measured daily using the Ammonia Assay Kit (Sigma-Aldrich, St. Louis, MI, USA).

However, BP-3 in the tested medium was not quantified in the chronic toxicity test of *M. macrocopa*, because media were completely exchanged every other day. 

#### 2.1.7. Statistical Analysis

Data are presented as the mean ± standard deviation (SD) and were statistically compared with the solvent control by one-way analysis of variance (ANOVA) post hoc Dunnett’s multiple comparison test using GraphPad Prism 5 (Ver. 5.01, GraphPad Software Inc., San Diego, CA, USA). Statistical significance was defined as *p* < 0.05. 

### 2.2. Effect Assessment

#### 2.2.1. Toxicity Data Collection

In this study, a total of five chronic aquatic toxicity datasets (e.g., NOEC, 20% effect concentration (EC20) and 10% inhibition concentration (IC10)) covering four taxonomic classes (fish, crustaceans, green algae, and cyanobacteria) were collected through a literature survey (Table 2). We assessed the reliability of the collected toxicity data using ToxRTool, a reliability assessment Excel spreadsheet, according to the Klimisch method. The Klimisch reliability score of each dataset was assigned as 2 (reliable with restrictions), meaning that those data were sufficiently reliable to be used for PNEC derivation. The reliability scoring sheets of each toxicity dataset obtained from the literature survey and in this study are shown in Table S1 of the Supplementary Materials.

#### 2.2.2. Predicted No-Effect Concentration (PNEC) Derivation

A total of eight chronic toxicity datasets (five from the literature survey plus three from toxicity testing) (Table 2) were used to derive the PNEC of BP-3. The PNEC value was derived by fitting the chronic NOEC, IC10 or EC20 value of each species to a linearized log-normal distribution using the SSD Curve Generator software developed by the USEPA. The PNEC value was determined at hazardous concentrations for 5% of species (HC5). Since NOEC values cannot be determined for *P. subcapitata*, we estimated the NOEC value (0.18 mg/L) by dividing the lowest observed effect concentration (LOEC, 0.45 mg/L) by 2.5 [20].

## 3. Results

### 3.1. Acute Toxicity

Growth inhibition (for *P. subcapitata*), immobility (for *M. macrocopa*), and survival rate (for *C. carpio*) were estimated for each aqueous organism exposed to BP-3, and EC50 or LC50 were calculated using CETIS software. The 72 h-EC50 for *P. subcapitata* was 1.82 (1.04–2.34, 95% CI) mg/L, the 48 h-EC50 for *M. macrocopa* was 4.59 (3.29–6.41, 95% CI) mg/L, and the 96 h-LC50 for *C. carpio* was 6.62 (4.68–9.35, 95% CI) mg/L (Figure S1).

### 3.2. Chronic Toxicity

In the chronic toxicity test of *M. macrocopa* exposed to BP-3 for 14 days, mortality was observed at 1 mg/L. The first day of reproduction, brood size, and the number of young per female were quantified; no toxicity effect other than lethality was observed at the tested concentrations. The chronic toxicity values (LOEC and NOEC) are presented in Table 3.

**Figure 1:**
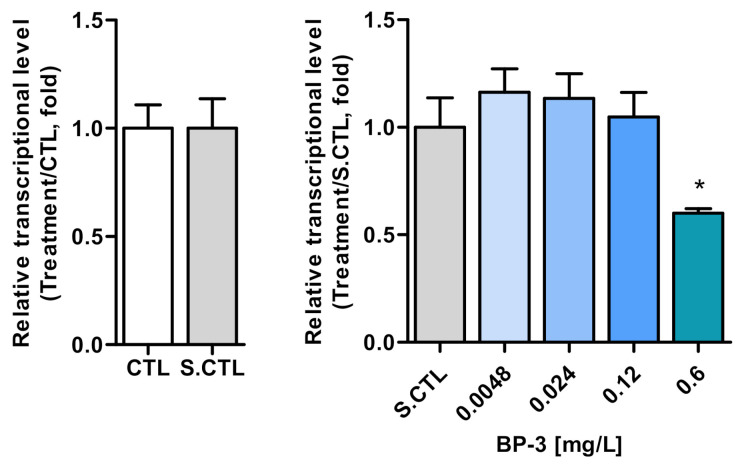
Relative transcriptional level of vtg1 in the liver of *Cyprinus carpio* exposed to benzophenone-3 for 21 days. No difference between blank and solvent controls were shown. All treatments were compared to a solvent control. Data are presented as mean ± SD and were statistically analyzed using a one-way ANOVA post hoc Dunnett’s multiple comparison test (*p < 0.05*): * *p* < 0.05.

**Figure 2:**
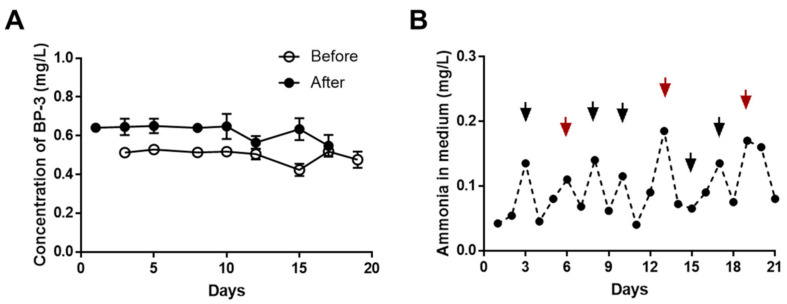
Concentrations of benzophenone-3 (BP-3) (**A**) and ammonia (**B**) during chronic toxicity test (21 days). (**A**) BP-3 was measured three times a week whenever the medium was changed. The concentrations of BP-3 were measured as 0.57–0.65 mg/L after replacing the culture medium (black circle) and as 0.43–0.53 mg/L before replacement (outlined circle). (**B**) Ammonia was measured daily. Black arrows indicate when half of the culture medium was exchanged, and red arrows indicate when the entire culture medium was exchanged.

During the chronic toxicity test of *C. carpio* exposed to BP-3 for 21 days, abnormal behaviors and mortality were not observed. The transcriptional levels of vitellogenin 1 were remarkably decreased at 0.6 mg/L (Figure 1). Based on this result, the chronic toxicity values (LOEC and NOEC) were determined and are summarized in Table 3. During the chronic toxicity test of *C. carpio* exposed to BP-3, the actual concentrations of BP-3 and ammonia were measured whenever the culture medium was changed (three times a week); these are presented in Figure 2. After replacing the culture medium, the concentration of BP-3 was measured as 0.57–0.65 mg/L, whereas the concentration of BP-3 before replacement was measured as 0.43–0.53 mg/L (Figure 2A). This shows that the concentration of BP-3 decreased by about 18–25% over 2–3 days. Ammonia concentration was measured as 0.04–0.18 mg/L during the test (Figure 2B), which was significantly lower than the ammonia toxicity value for *C. carpio* [27].

### 3.3. PNEC Derivation

**Figure 3:**
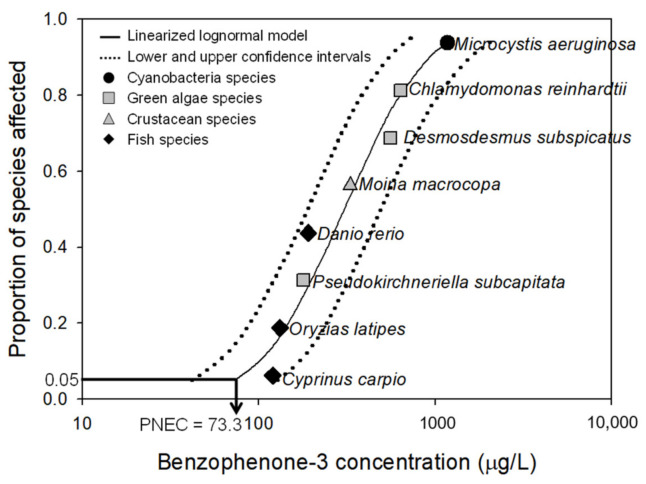
Species sensitivity distribution (SSD) for the chronic toxicity of benzophenone-3. Predicted no-effect concentration (PNEC) was set at the 5% hazardous concentration (HC5).

Chronic toxicity data on eight species (Table 3) covering four taxonomic classes (i.e., fish, crustacean, green algae and cyanobacteria) were fitted to a linearized log-normal distribution (Figure 3). The chronic toxicity data ranged from 120 to 1170 μg/L. The most sensitive toxicity value was the NOEC of 120 μg/L for the reproductive effect on C. carpio. The derived PNEC value of BP-3 was 73.3 μg/L (prediction interval: 39.8~132.1 μg/L).

The quality and quantity of chronic toxicity data used in this study satisfied the minimum requirements for the PNEC derivation of BP-3. Eight chronic toxicity data points covering four taxonomic classes, of which the Klimisch reliability scores were assigned as 1 or 2, were used. Further, because the fit of the SSD to the toxicity data was good (r^2^ > 0.9), the reliability of the PNEC value derived in this study is high enough to be used for the aquatic risk assessment of BP-3.

**Figure 4:**
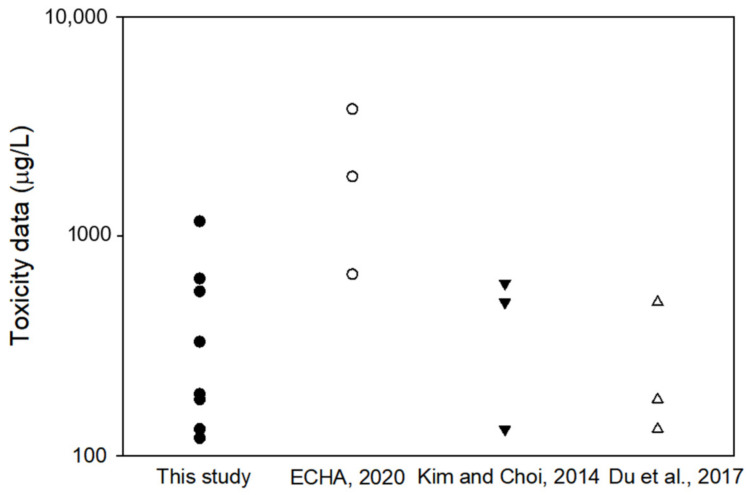
Toxicity data used for the derivation of predicted no-effect concentration (PNEC) of benzophenone-3 in this and three earlier studies (ECHA, 2020 [18]; Kim and Choi, 2014 [4]; and Du et al., 2017 [12]).

The derived PNEC in this study (73.3 μg/L) was 40~108 times higher than the existing PNECs (0.67~1.8 μg/L). Because the chronic toxicity value range (102~1170 μg/L) used for the PNEC derivation in this study was not significantly different from that used in earlier studies (Figure 4), it is reasonable to think that the less conservative PNEC value is derived mainly due to the different PNEC derivation methodology (i.e., AF vs SSD approach). It is generally regarded that the AF approach induces a more conservative PNEC value than the SSD approach if the same toxicity data are used [28,29]. For example, Hahn et al. compared the PNEC values of ethylene glycol, trichloroethylene and Cu derived using both the AF and SSD approaches, and concluded that the SSD approach gave less variable PNECs [29].

## 4. Discussion

Setting a reliable PNEC value plays a key role in ecological risk assessment. If a set PNEC value is much higher than true toxicity value, then the risk may be underestimated so it may concluded that no risk management action is necessary even if the chemical concentration is high enough to pose a threat to ecological organisms. In contrast, if the set PNEC value is too conservative, it would be concluded that the risk management actions are required even in areas where no actual risk is suspected (i.e., risk is overestimated if PNEC value is too conservative). The SSD approach is recognized as a potential option that can derive more statistically reliable PNEC values than the AF approach because the SSD approach derives a PNEC value using various toxicity data while the AF approach derives a PNEC value using only one piece of toxicity data (i.e., the lowest toxicity value).

Our study revealed that existing PNEC values derived using AF approaches were too conservative, so risk would be overestimated. Kim and Choi determined PNEC values of BP-3 at 1.32 μg/L by using an AF approach and argued that the concentration of BP-3 in some hotspots (e.g., wastewater influents of which BP-3 concentration is as high as 10.4 μg/L) exceeded PNEC values [4]. However, such a concentration is one order of magnitude lower than the most sensitive toxicity value of BP-3 (120 μg/L), and thus does not seem to provoke adverse effects to aquatic organisms. This implies that existing PNEC values may unnecessarily overestimate the risk posed by BP-3. Du et al. also acknowledged the possibility of risk overestimation when the PNEC value derived with an AF approach is applied [12]. In contrast, if the PNEC value derived in this study is applied, it would be determined that the BP-3 concentration is far lower than PNEC value even in hotspots, so that no action for BP-3 management is warranted.

The SSD approach has a strong advantage in that it can reduce uncertainty by increasing toxicity data if the quantity and quality of toxicity data meet the requirements for PNEC derivation. Therefore, we believe that the PNEC value derived in this study is highly reliable and can eliminate the possibility of excessive risk overestimation, and thus, existing PNEC values derived from AF approaches should be revised to those derived from an SSD approach.

## 5. Conclusions

In conclusion, we suggested the freshwater PNEC value of BP-3 derived using a SSD approach, rather than AF approach, based on the chronic toxicity data obtained from the experiment and literature survey, because the former is more statistically reliable for PNEC value derivation than the latter, which often provides an overestimated risk. To the best of our knowledge, this is the first suggestion of the freshwater PNEC of BP-3 using the SSD approach, which seems to be more statistically powerful than the AF approach. We hope that our suggestion contributes to the more efficient management of BP-3 risk.

## Figures and Tables

**Table 1:** The maximum concentration (C0) of BP-3 in each medium (ATCC, M4 and fish water) and the concentrations used in acute and chronic toxicity tests.

Species	C0 (mg/L)	Tested Concentrations (mg/L)	
		Acute Tests ^1^	Chronic Tests
*Pseudokirchneriella subcapitata*	17.17 ± 0.42	0.45, 1.13, 2.83, 7.08, 17.71	NA ^2^
*Moina macrocopa*	23.55 ± 0.38	0.52, 1.00, 2.15, 5.04, 8.25	0.012, 0.037, 0.11, 0.33, 1
*Cyprinus carpio*	37.40 ± 1.02	0.73, 1.18, 2.17, 4.68, 9.35	0.0048, 0.024, 0.12, 0.6

^1^ Concentrations used in the acute toxicity teste are diluted from C0. ^2^ NA means not analyzed.

**Table 2:** The chronic toxicity data used for the derivation of predicted no-effect concentration (PNEC) of benzophenone-3 (BP-3) in this study.

Species	Taxonomic Class	Exposure Duration (Days)	Effect	Endpoint	Toxicity Value (μg/L)	Source	Reliability Category
*Chlamydomonas reinhardtii*	green algae	10	chl-a reduction	EC20 ^a^	640	[26]	2
*Cyprinus carpio*	fish	21	vitellogenin reduction	NOEC ^b^	120	this study	2
*Danio rerio*	fish	60	skewing of phenotypic sex ratio	NOEC	191	[16]	2
*Desmodesmus subspicatus*	green algae	3	growth inhibition	IC10 ^c^	560	[13]	2
*Microcystis aeruginosa*	cyanobacteria	10	chl-a reduction	EC20	1170	[26]	2
*Moina macrocopa*	crustacean	3 brood	lethality	NOEC	330	this study	2
*Oryzias latipes*	fish	21	reproduction	NOEC	132	[15]	2
*Pseudokirchneriella subcapitata*	green algae	3	growth	NOEC	180	this study	2

^a^ 20% effect concentration. ^b^ no observed effect concentration. ^c^ 10% inhibition concentration.

**Table 3:** Lowest observed effect concentration (LOEC) and no observed effect concentration (NOEC) of benzophenone-3 to *Moina macrocopa* and *Cyprinus carpio* in the chronic toxicity tests.

Toxicity Data (mg/L)	*M. Macrocopa*				*C. Carpio*
	Mortality	First Day of Reproduction	Number of Young Per Female	Blood Size	Vtg 1
LOEC	1	>1	>1	>1	0.6
NOEC	0.33	1	1	1	0.12

## Supplementary Materials

The following are available online at https://www.mdpi.com/article/10.3390/ijerph18073650/s1, Figure S1: Growth inhibition, immobility, and survival rate of *Pseudokirchneriella subcapitata* (A), *Moina macrocopa* (B), and *Cyprinus carpio* (C) exposed to benzophenone-3, respectively; Table S1: Reliability assessment sheets of toxicity data used for the PNEC derivation in this study.
