# Supplementary material for: A Novel Approach to Derive the Predicted No-Effect Concentration (PNEC) of Benzophenone-3 (BP-3) Using the Species Sensitivity Distribution (SSD) Method: Suggestion of a New PNEC Value for BP-3
Source: Int J Environ Res Public Health. 2021 Mar 31;18(7):3650. doi: 10.3390/ijerph18073650 (PMC8037607; doi:10.3390/ijerph18073650)
Supplement: Supplementary file 1 [file ijerph-18-03650-s001.pdf]

Supplementary Data

A novel approach to derive the predicted no-effect concentration (PNEC) of benzophenone-3 (BP-3) using the species sensitivity distribution (SSD) method: Suggestion of a new PNEC value for BP-3

Jae-Woong Jung [1,†], Jae Soon Kang [2,†], Jinsoo Choi [3] and June-Woo Park [3,4,*]

[1] Center for Defense Acquisition and Requirements Analysis, Korea Institute for Defense Analyses, Seoul 02455, Korea; jwjung@kida.re.kr
[2] Department of Anatomy and Convergence Medical Science, Institute of Health Science, Bio Anti-aging Medical Research Center, Gyeongsang National University Medical School, Jinju 52727, Korea; jskang@gnu.ac.kr
[3] Department of Environmental Toxicology and Chemistry, Korea Institute of Toxicology, Jinju 52834, Korea; jschoi1020@kitox.re.kr
[4] Human and Environmental Toxicology Program, Korea University of Science and Technology (UST),
Daejeon 34113, Korea
* Correspondence: jwpark@kitox.re.kr; Tel.: +82-55-750-3833
† These authors contributed equally to this work

Number of Figure: 1

Number of Table: 1

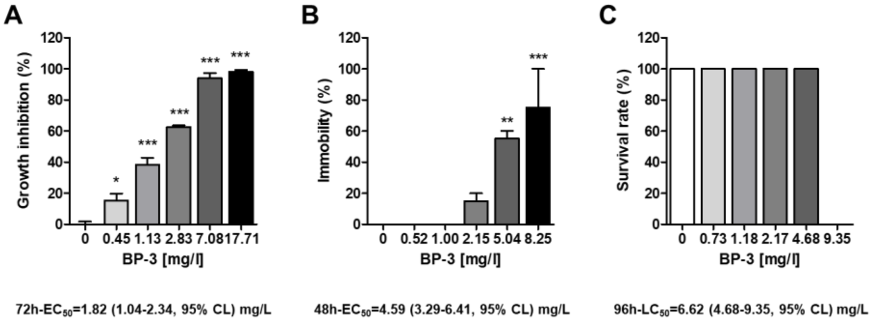

Figure S1. Growth inhibition, immobility, and survival rate of *Pseudokirchneriella subcapitata* (A), *Moina macrocopa* (B), and *Cyprinus carpio* (C) exposed to benzophenone-3, respectively. Data are presented as mean ± SD and were statistically analyzed using a one-way ANOVA post hoc Dunnett's multiple comparison test ($p < 0.05$): $*p < 0.05$, $**p < 0.01$ and $***p < 0.001$.

Table S1. Reliability assessment sheets of toxicity data used for the PNEC derivation in this study

(a) *Chlamydomonas reinhardtii*

**Reliability assessment of in vivo toxicity studies**			
**Study under evaluation**			
Authors:			
	Mao et al.		
Titel:			
	Effects of benzophenone-3 on the green alga Chlamydomonas reinhardtii and the cyanobacterium Microcystis aeruginosa		
Testing facility, year, sponsor, study no. or bibliographic reference:			
	Aquatic Toxicology 193 (2017), 1-8		
Explanations are available for most criteria and show up, when the cursor is moved over the criteria field. Please read carefully! Red criteria: the maximum score is needed for these criteria to achieve reliability category 1 or 2 (see worksheet Explanations): Please evaluate with special care!			
**Criteria**			**Evaluator's explanations, comments on criteria, etc.**
**No.**	**Criteria Group I: Test substance identification**	**Score**	
1	Was the test substance identified?	1	Oxybenzone
2	Is the purity of the substance given?		
3	Is information on the source/origin of the substance given?	1	Sigma
4	Is all information on the nature and/or physico-chemical properties of the test item given, which you deem indispensable for judging the data (see explanation for examples)?	0	
		2	
	**Criteria Group II: Test organism characterisation**		
5	Is the species given?	1	Chlamydomonas reinhardtii
6	Is the sex of the test organism given?	1	algae
7	Is information given on the strain of test animals plus, if considered necessary to judge the study, other specifications (see explanation for examples)?	1	NIES 2463
8	Is age or body weight of the test organisms at the start of the study given?	1	algae
9	For repeated dose toxicity studies only (give point for other study types): Is information given on the housing or feeding conditions?	1	Not repeated dose toxicity study
		5	
	**Criteria Group III: Study design description**		
10	Is the administration route given?	1	Exposure by contaminated water
11	Are doses administered or concentrations in application media given?	1	0.01, 0.1, 1, 10, 100, 1000, 2000, 3000, 4000, 5000 ug/L
12	Are frequency and duration of exposure as well as time-points of observations explained?	1	10 days
13	Were negative (where required) and positive controls (where required) included (give point also, when absent but not required, see explanations for study types and their respective requirements on controls)?	1	blank control
14	Is the number of animals (in case of experimental human studies: number of test persons) per group given?	1	500000 cells/mL
15	Are sufficient details of the administration scheme given to judge the study (see explanation for examples)?		
16	For inhalation studies and repeated dose toxicity studies only (give point for other study types): Were achieved concentrations analytically verified or was stability of the test substance otherwise ensured or made plausible?	1	Not inhalation study
		6	
	**Criteria Group IV: Study results documentation**		
17	Are the study endpoint(s) and their method(s) of determination clearly described?	1	chl-a content decrease
18	Is the description of the study results for all endpoints investigated transparent and complete?		
19	Are the statistical methods applied for data analysis given and applied in a transparent manner (give also point, if not necessary/applicable, see explanations)?	0	
		1	
	**Criteria Group V: Plausibility of study design and results**		
20	Is the study design chosen appropriate for obtaining the substance-specific data aimed at (see explanations for details)?	1	
21	Are the quantitative study results reliable (see explanations for arguments)?		
		1	
			**WARNING: check for unprocessed criteria! (for each criterium a score has to be selected)**
		15	
	**A Numerical result leads to initial Category:**	**2**	
	**B Checking red scores leads to revised Category:**	**2**	
	**C Evaluator's proposal: Category:**		
	**D Justification in case evaluator deviates from B:**		

(b) *Cyprinus carpio*

Reliability assessment of in vivo toxicity studies			
**Study under evaluation**			
Authors:			
Titel:			
Testing facility, year, sponsor, study no. or bibliographic reference:			
Explanations are available for most criteria and show up, when the cursor is moved over the criteria field. Please read carefully! Red criteria: the maximum score is needed for these criteria to achieve reliability category 1 or 2 (see worksheet Explanations): Please evaluate with special care!			
**Criteria**			Evaluator's explanations, comments on criteria, etc.
**No.**	**Criteria Group I: Test substance identification**	**Score**	
1	Was the test substance identified?	1	Oxybenzone
2	Is the purity of the substance given?	1	98%
3	Is information on the source/origin of the substance given?	1	Sigma-Aldrich
4	Is all information on the nature and/or physico-chemical properties of the test item given, which you deem indispensable for judging the data (see explanation for examples)?	0	
		3	
	**Criteria Group II: Test organism characterisation**		
5	Is the species given?	1	Cyprinus carpio
6	Is the sex of the test organism given?	0	
7	Is information given on the strain of test animals plus, if considered necessary to judge the study, other specifications (see explanation for examples)?	0	
8	Is age or body weight of the test organisms at the start of the study given?	0	
9	For repeated dose toxicity studies only (give point for other study types): Is information given on the housing or feeding conditions?	1	Not repeated dose toxicity study
		2	
	**Criteria Group III: Study design description**		
10	Is the administration route given?	1	Exposure by contaminated water
11	Are doses administered or concentrations in application media given?	1	0.0048, 0.024, 0.12, 0.6 mg/L
12	Are frequency and duration of exposure as well as time-points of observations explained?	1	21 days
13	Were negative (where required) and positive controls (where required) included (give point also, when absent but not required, see explanations for study types and their respective requirements on controls)?	1	Blank control
14	Is the number of animals (in case of experimental human studies: number of test persons) per group given?	1	5 fish
15	Are sufficient details of the administration scheme given to judge the study (see explanation for examples)?		
16	For inhalation studies and repeated dose toxicity studies only (give point for other study types): Were achieved concentrations analytically verified or was stability of the test substance otherwise ensured or made plausible?	1	Not inhalation study
		6	
	**Criteria Group IV: Study results documentation**		
17	Are the study endpoint(s) and their method(s) of determination clearly described?	1	Decrease in transcriptional levels of estrogenic genes
18	Is the description of the study results for all endpoints investigated transparent and complete?	0	
19	Are the statistical methods applied for data analysis given and applied in a transparent manner (give also point, if not necessary/applicable, see explanations)?	0	
		1	
	**Criteria Group V: Plausibility of study design and results**		
20	Is the study design chosen appropriate for obtaining the substance-specific data aimed at (see explanations for details)?	1	
21	Are the quantitative study results reliable (see explanations for arguments)?		
		1	
			**WARNING: check for unprocessed criteria! (for each criterium a score has to be selected)**
		13	
	**A Numerical result leads to initial Category:**	**2**	
	**B Checking red scores leads to revised Category:**	**2**	
	**C Evaluator's proposal: Category:**		
	**D Justification in case evaluator deviates from B:**		

(c) *Danio rerio*

**Reliability assessment of in vivo toxicity studies**			
**Study under evaluation**			
Authors:			
	Kinnberg et al.		
Titel:			
	Endocrine-disrupting effect of the untraviolate filter benzophenone-3 in zebrafish, Danio rerio		
Testing facility, year, sponsor, study no. or bibliographic reference:			
	Environ. Toxicol. Chem. 34 (2015), 2833-2840		
Explanations are available for most criteria and show up, when the cursor is moved over the criteria field. Please read carefully! Red criteria: the maximum score is needed for these criteria to achieve reliability category 1 or 2 (see worksheet Explanations): Please evaluate with special care!			
**Criteria**			**Evaluator's explanations, comments on criteria, etc.**
**No.**	**Criteria Group I: Test substance identification**	**Score**	
1	Was the test substance identified?	1	Oxybenzone
2	Is the purity of the substance given?		
3	Is information on the source/origin of the substance given?	1	Rathburn Chemicals
4	Is all information on the nature and/or physico-chemical properties of the test item given, which you deem indispensable for judging the data (see explanation for examples)?	0	
		2	
	**Criteria Group II: Test organism characterisation**		
5	Is the species given?	1	Danio rerio
6	Is the sex of the test organism given?	1	larvae
7	Is information given on the strain of test animals plus, if considered necessary to judge the study, other specifications (see explanation for examples)?	0	
8	Is age or body weight of the test organisms at the start of the study given?	1	1 dpf
9	For repeated dose toxicity studies only (give point for other study types): Is information given on the housing or feeding conditions?	1	Not repeated dose toxicity study
		4	
	**Criteria Group III: Study design description**		
10	Is the administration route given?	1	Exposure by contaminated water
11	Are doses administered or concentrations in application media given?	1	0, 191, 388, 470
12	Are frequency and duration of exposure as well as time-points of observations explained?	1	60 dpf
13	Were negative (where required) and positive controls (where required) included (give point also, when absent but not required, see explanations for study types and their respective requirements on controls)?	1	blank control, solvent control
14	Is the number of animals (in case of experimental human studies: number of test persons) per group given?	1	30
15	Are sufficient details of the administration scheme given to judge the study (see explanation for examples)?		
16	For inhalation studies and repeated dose toxicity studies only (give point for other study types): Were achieved concentrations analytically verified or was stability of the test substance otherwise ensured or made plausible?	1	Not inhalation study
		6	
	**Criteria Group IV: Study results documentation**		
17	Are the study endpoint(s) and their method(s) of determination clearly described?	1	skewing of the phenotypic sex ratio
18	Is the description of the study results for all endpoints investigated transparent and complete?		
19	Are the statistical methods applied for data analysis given and applied in a transparent manner (give also point, if not necessary/applicable, see explanations)?	0	
		1	
	**Criteria Group V: Plausibility of study design and results**		
20	Is the study design chosen appropriate for obtaining the substance-specific data aimed at (see explanations for details)?	1	
21	Are the quantitative study results reliable (see explanations for arguments)?		
		1	
			**WARNING: check for unprocessed criteria! (for each criterium a score has to be selected)**
		14	
	**A Numerical result leads to initial Category:**	**2**	
	**B Checking red scores leads to revised Category:**	**2**	
	**C Evaluator's proposal: Category:**		
	**D Justification in case evaluator deviates from B:**		

(d) *Desmodesmus subspicatus*

**Reliability assessment of in vivo toxicity studies**			
**Study under evaluation**			
Authors:			
	Sieratowicz et al.		
Titel:			
	Acute and chronic toxicity of four frequently used UV filter substances for Desmodesmus subspicatus and Daphnia magna		
Testing facility, year, sponsor, study no. or bibliographic reference:			
	J. Environ. Sci. Heal., Part A 46 (2011), 1311-1319		
Explanations are available for most criteria and show up, when the cursor is moved over the criteria field. Please read carefully! Red criteria: the maximum score is needed for these criteria to achieve reliability category 1 or 2 (see worksheet Explanations): Please evaluate with special care!			
**Criteria**			**Evaluator's explanations, comments on criteria, etc.**
**No.**	**Criteria Group I: Test substance identification**	**Score**	
1	Was the test substance identified?	1	Oxybenzone
2	Is the purity of the substance given?	0	
3	Is information on the source/origin of the substance given?	1	Sigma
4	Is all information on the nature and/or physico-chemical properties of the test item given, which you deem indispensable for judging the data (see explanation for examples)?	0	
		2	
	**Criteria Group II: Test organism characterisation**		
5	Is the species given?	1	Desmodesmus subspicatus
6	Is the sex of the test organism given?	1	algal test
7	Is information given on the strain of test animals plus, if considered necessary to judge the study, other specifications (see explanation for examples)?	0	
8	Is age or body weight of the test organisms at the start of the study given?	1	algal test
9	For repeated dose toxicity studies only (give point for other study types): Is information given on the housing or feeding conditions?	1	Not repeated dose toxicity study
		4	
	**Criteria Group III: Study design description**		
10	Is the administration route given?	1	Exposure by contaminated water
11	Are doses administered or concentrations in application media given?	1	0.06, 0.13, 0.25, 0.5, 1 ug/L
12	Are frequency and duration of exposure as well as time-points of observations explained?	1	24, 48, 72 hours
13	Were negative (where required) and positive controls (where required) included (give point also, when absent but not required, see explanations for study types and their respective requirements on controls)?	1	solvent control
14	Is the number of animals (in case of experimental human studies: number of test persons) per group given?	1	50000 cells per replicate
15	Are sufficient details of the administration scheme given to judge the study (see explanation for examples)?		
16	For inhalation studies and repeated dose toxicity studies only (give point for other study types): Were achieved concentrations analytically verified or was stability of the test substance otherwise ensured or made plausible?	1	Not inhalation study
		6	
	**Criteria Group IV: Study results documentation**		
17	Are the study endpoint(s) and their method(s) of determination clearly described?	1	specific growth rate
18	Is the description of the study results for all endpoints investigated transparent and complete?		
19	Are the statistical methods applied for data analysis given and applied in a transparent manner (give also point, if not necessary/applicable, see explanations)?	0	
		1	
	**Criteria Group V: Plausibility of study design and results**		
20	Is the study design chosen appropriate for obtaining the substance-specific data aimed at (see explanations for details)?	1	
21	Are the quantitative study results reliable (see explanations for arguments)?		
		1	
			**WARNING: check for unprocessed criteria! (for each criterium a score has to be selected)**
		14	
	**A Numerical result leads to initial Category:**	**2**	
	**B Checking red scores leads to revised Category:**	**2**	
	**C Evaluator's proposal: Category:**		
	**D Justification in case evaluator deviates from B:**		

(e) *Microcystis aeruginosa*

Reliability assessment of in vivo toxicity studies			
**Study under evaluation**			
Authors:			
	Mao et al.		
Titel:			
	Effects of benzophenone-3 on the green alga Chlamydomonas reinhardtii and the cyanobacterium Microcystis aeruginosa		
Testing facility, year, sponsor, study no. or bibliographic reference:			
	Aquatic Toxicology 193 (2017), 1-8		
Explanations are available for most criteria and show up, when the cursor is moved over the criteria field. Please read carefully! Red criteria: the maximum score is needed for these criteria to achieve reliability category 1 or 2 (see worksheet Explanations): Please evaluate with special care!			
**Criteria**			**Evaluator's explanations, comments on criteria, etc.**
**No.**	**Criteria Group I: Test substance identification**	**Score**	
1	Was the test substance identified?	1	Oxybenzone
2	Is the purity of the substance given?		
3	Is information on the source/origin of the substance given?	1	Sigma
4	Is all information on the nature and/or physico-chemical properties of the test item given, which you deem indispensable for judging the data (see explanation for examples)?	0	
		2	
	**Criteria Group II: Test organism characterisation**		
5	Is the species given?	1	Microcystis aeruginosa
6	Is the sex of the test organism given?	1	cyanobacteria
7	Is information given on the strain of test animals plus, if considered necessary to judge the study, other specifications (see explanation for examples)?	1	NIES 843
8	Is age or body weight of the test organisms at the start of the study given?	1	cyanobacteria
9	For repeated dose toxicity studies only (give point for other study types): Is information given on the housing or feeding conditions?	1	Not repeated dose toxicity study
		5	
	**Criteria Group III: Study design description**		
10	Is the administration route given?	1	Exposure by contaminated water
11	Are doses administered or concentrations in application media given?	1	0.01, 0.1, 1, 10, 100, 1000, 2000, 3000, 4000, 5000 ug/L
12	Are frequency and duration of exposure as well as time-points of observations explained?	1	10 days
13	Were negative (where required) and positive controls (where required) included (give point also, when absent but not required, see explanations for study types and their respective requirements on controls)?	1	blank control
14	Is the number of animals (in case of experimental human studies: number of test persons) per group given?	1	1000000 cells/mL
15	Are sufficient details of the administration scheme given to judge the study (see explanation for examples)?		
16	For inhalation studies and repeated dose toxicity studies only (give point for other study types): Were achieved concentrations analytically verified or was stability of the test substance otherwise ensured or made plausible?	1	Not inhalation study
		6	
	**Criteria Group IV: Study results documentation**		
17	Are the study endpoint(s) and their method(s) of determination clearly described?	1	chl-a content decrease
18	Is the description of the study results for all endpoints investigated transparent and complete?		
19	Are the statistical methods applied for data analysis given and applied in a transparent manner (give also point, if not necessary/applicable, see explanations)?	0	
		1	
	**Criteria Group V: Plausibility of study design and results**		
20	Is the study design chosen appropriate for obtaining the substance-specific data aimed at (see explanations for details)?	1	
21	Are the quantitative study results reliable (see explanations for arguments)?		
		1	
			**WARNING: check for unprocessed criteria! (for each criterium a score has to be selected)**
		15	
	**A Numerical result leads to initial Category:**	**2**	
	**B Checking red scores leads to revised Category:**	**2**	
	**C Evaluator's proposal: Category:**		
	**D Justification in case evaluator deviates from B:**		

(f) *Moina macrocopa*

	**Reliability assessment of in vivo toxicity studies**		
	**Study under evaluation**		
	Authors:		
	Titel:		
	Testing facility, year, sponsor, study no. or bibliographic reference:		
	Explanations are available for most criteria and show up, when the cursor is moved over the criteria field. Please read carefully! Red criteria: the maximum score is needed for these criteria to achieve reliability category 1 or 2 (see worksheet Explanations): Please evaluate with special care!		
	**Criteria**		**Evaluator's explanations, comments on criteria, etc.**
**No.**	**Criteria Group I: Test substance identification**	**Score**	
1	Was the test substance identified?	1	Oxybenzone
2	Is the purity of the substance given?	1	98%
3	Is information on the source/origin of the substance given?	1	Sigma-Aldrich
4	Is all information on the nature and/or physico-chemical properties of the test item given, which you deem indispensable for judging the data (see explanation for examples)?	0	
		3	
	**Criteria Group II: Test organism characterisation**		
5	Is the species given?	1	Moina macrocopa
6	Is the sex of the test organism given?	1	Female
7	Is information given on the strain of test animals plus, if considered necessary to judge the study, other specifications (see explanation for examples)?	0	
8	Is age or body weight of the test organisms at the start of the study given?	1	<24 hours
9	For repeated dose toxicity studies only (give point for other study types): Is information given on the housing or feeding conditions?	1	Not repeated dose toxicity study
		4	
	**Criteria Group III: Study design description**		
10	Is the administration route given?	1	Exposure by contaminated water
11	Are doses administered or concentrations in application media given?	1	0.012, 0.037, 0.11, 0.33, 1 mg/L
12	Are frequency and duration of exposure as well as time-points of observations explained?	1	14 days
13	Were negative (where required) and positive controls (where required) included (give point also, when absent but not required, see explanations for study types and their respective requirements on controls)?	1	Blank control
14	Is the number of animals (in case of experimental human studies: number of test persons) per group given?	1	10 per group
15	Are sufficient details of the administration scheme given to judge the study (see explanation for examples)?		
16	For inhalation studies and repeated dose toxicity studies only (give point for other study types): Were achieved concentrations analytically verified or was stability of the test substance otherwise ensured or made plausible?	1	Not inhalation study
		6	
	**Criteria Group IV: Study results documentation**		
17	Are the study endpoint(s) and their method(s) of determination clearly described?	1	Reproduction
18	Is the description of the study results for all endpoints investigated transparent and complete?	0	
19	Are the statistical methods applied for data analysis given and applied in a transparent manner (give also point, if not necessary/applicable, see explanations)?	0	
		1	
	**Criteria Group V: Plausibility of study design and results**		
20	Is the study design chosen appropriate for obtaining the substance-specific data aimed at (see explanations for details)?	1	
21	Are the quantitative study results reliable (see explanations for arguments)?		
		1	
			**WARNING: check for unprocessed criteria! (for each criterium a score has to be selected)**
		15	
	**A Numerical result leads to initial Category:**	**2**	
	**B Checking red scores leads to revised Category:**	**2**	
	**C Evaluator's proposal: Category:**		
	**D Justification in case evaluator deviates from B:**		

(g) *Oryzias latipes*

**Reliability assessment of in vivo toxicity studies**			
**Study under evaluation**			
Authors:			
	Coronado et al.		
Titel:			
	Estrogenic activity and reproductive effects of the UV-filter oxybenzone (2-hydroxy-4-methoxyphenyl-methanone) in fish		
Testing facility, year, sponsor, study no. or bibliographic reference:			
	Aquatic Toxicology 90 (2002), 182-187		
Explanations are available for most criteria and show up, when the cursor is moved over the criteria field. Please read carefully! Red criteria: the maximum score is needed for these criteria to achieve reliability category 1 or 2 (see worksheet Explanations): Please evaluate with special care!			
**Criteria**			**Evaluator's explanations, comments on criteria, etc.**
**No.**	**Criteria Group I: Test substance identification**	**Score**	
1	Was the test substance identified?	1	Oxybenzone
2	Is the purity of the substance given?	1	99%
3	Is information on the source/origin of the substance given?	1	Sigma
4	Is all information on the nature and/or physico-chemical properties of the test item given, which you deem indispensable for judging the data (see explanation for examples)?	0	
		3	
	**Criteria Group II: Test organism characterisation**		
5	Is the species given?	1	Oryzias latipes
6	Is the sex of the test organism given?	1	both males and females
7	Is information given on the strain of test animals plus, if considered necessary to judge the study, other specifications (see explanation for examples)?	0	
8	Is age or body weight of the test organisms at the start of the study given?	1	3 months
9	For repeated dose toxicity studies only (give point for other study types): Is information given on the housing or feeding conditions?	1	Not repeated dose toxicity study
		4	
	**Criteria Group III: Study design description**		
10	Is the administration route given?	1	Exposure by contaminated water
11	Are doses administered or concentrations in application media given?	1	0, 16, 132, 620 ug/L
12	Are frequency and duration of exposure as well as time-points of observations explained?	1	1, 2, 3 weeks
13	Were negative (where required) and positive controls (where required) included (give point also, when absent but not required, see explanations for study types and their respective requirements on controls)?	1	Blank control and solvent control
14	Is the number of animals (in case of experimental human studies: number of test persons) per group given?	1	2 males and 2 females in each replicate
15	Are sufficient details of the administration scheme given to judge the study (see explanation for examples)?		
16	For inhalation studies and repeated dose toxicity studies only (give point for other study types): Were achieved concentrations analytically verified or was stability of the test substance otherwise ensured or made plausible?	1	Not inhalation study
		6	
	**Criteria Group IV: Study results documentation**		
17	Are the study endpoint(s) and their method(s) of determination clearly described?	1	vitellogenin and reproduction
18	Is the description of the study results for all endpoints investigated transparent and complete?		
19	Are the statistical methods applied for data analysis given and applied in a transparent manner (give also point, if not necessary/applicable, see explanations)?	0	
		1	
	**Criteria Group V: Plausibility of study design and results**		
20	Is the study design chosen appropriate for obtaining the substance-specific data aimed at (see explanations for details)?	1	
21	Are the quantitative study results reliable (see explanations for arguments)?		
		1	
			**WARNING: check for unprocessed criteria! (for each criterium a score has to be selected)**
		15	
	**A Numerical result leads to initial Category:**	**2**	
	**B Checking red scores leads to revised Category:**	**2**	
	**C Evaluator's proposal: Category:**		
	**D Justification in case evaluator deviates from B:**		

(h) *Pseudokirchneriella subcapitata*

**Reliability assessment of in vivo toxicity studies**			
**Study under evaluation**			
Authors:			
Titel:			
Testing facility, year, sponsor, study no. or bibliographic reference:			
Explanations are available for most criteria and show up, when the cursor is moved over the criteria field. Please read carefully! Red criteria: the maximum score is needed for these criteria to achieve reliability category 1 or 2 (see worksheet Explanations): Please evaluate with special care!			
**Criteria**			**Evaluator's explanations, comments on criteria, etc.**
**No.**	**Criteria Group I: Test substance identification**	**Score**	
1	Was the test substance identified?	1	Oxybenzone
2	Is the purity of the substance given?	1	98%
3	Is information on the source/origin of the substance given?	1	Sigma-Aldrich
4	Is all information on the nature and/or physico-chemical properties of the test item given, which you deem indispensable for judging the data (see explanation for examples)?	0	
		3	
	**Criteria Group II: Test organism characterisation**		
5	Is the species given?	1	Pseudokirchneriella subcapitata
6	Is the sex of the test organism given?	1	Algal test
7	Is information given on the strain of test animals plus, if considered necessary to judge the study, other specifications (see explanation for examples)?	0	
8	Is age or body weight of the test organisms at the start of the study given?	0	
9	For repeated dose toxicity studies only (give point for other study types): Is information given on the housing or feeding conditions?	1	Not repeated dose toxicity study
		3	
	**Criteria Group III: Study design description**		
10	Is the administration route given?	1	Exposure by contaminated water
11	Are doses administered or concentrations in application media given?	1	0.45, 1.13, 2.83, 7.08, 17.71 mg/L
12	Are frequency and duration of exposure as well as time-points of observations explained?	1	3 days
13	Were negative (where required) and positive controls (where required) included (give point also, when absent but not required, see explanations for study types and their respective requirements on controls)?	1	Blank control
14	Is the number of animals (in case of experimental human studies: number of test persons) per group given?	1	Algal test
15	Are sufficient details of the administration scheme given to judge the study (see explanation for examples)?		
16	For inhalation studies and repeated dose toxicity studies only (give point for other study types): Were achieved concentrations analytically verified or was stability of the test substance otherwise ensured or made plausible?	1	Not inhalation study
		6	
	**Criteria Group IV: Study results documentation**		
17	Are the study endpoint(s) and their method(s) of determination clearly described?	1	Growth
18	Is the description of the study results for all endpoints investigated transparent and complete?	0	
19	Are the statistical methods applied for data analysis given and applied in a transparent manner (give also point, if not necessary/applicable, see explanations)?	0	
		1	
	**Criteria Group V: Plausibility of study design and results**		
20	Is the study design chosen appropriate for obtaining the substance-specific data aimed at (see explanations for details)?	1	
21	Are the quantitative study results reliable (see explanations for arguments)?		
		1	
			**WARNING: check for unprocessed criteria! (for each criterium a score has to be selected)**
		14	
	**A Numerical result leads to initial Category:**	**2**	
	**B Checking red scores leads to revised Category:**	**2**	
	**C Evaluator's proposal: Category:**		
	**D Justification in case evaluator deviates from B:**		
